# Hybrid Fourier-Derivative Analysis: An accurate and fast method for blood flow quantification in photoacoustic microscopy

**DOI:** 10.1016/j.pacs.2025.100761

**Published:** 2025-08-21

**Authors:** Zhuoying Wang, Ziang Feng, Song Hu

**Affiliations:** Department of Biomedical Engineering, Washington University in St. Louis, MO 63130, USA

**Keywords:** Blood flow quantification, Flow speed, Photoacoustic microscopy, Functional imaging, Signal processing

## Abstract

Photoacoustic microscopy (PAM) enables label-free, quantitative imaging of blood flow and oxygenation *in vivo*, offering critical insights into microvascular function and tissue metabolism. However, current flow quantification methods suffer from poor accuracy at extreme flow speeds and high computational costs. We present Hybrid Fourier-Derivative Analysis (HFDA), a new method based on frequency analysis of flow-induced modulations in photoacoustic amplitude. Compatible with standard raster scanning, HFDA adaptively integrates Fourier analysis for high-speed flow and derivative analysis for low-speed flow, achieving high accuracy and computational efficiency. Phantom studies validate the accuracy of HFDA across 0.2–20 mm/s, with errors typically less than 7 %. Compared to correlation-based methods, HFDA reduces computational time by 35-fold. *In vivo* demonstrations in mouse models of hypoxia and hypercapnia further underscore the potential of HFDA as a rapid and precise tool for blood flow quantification in functional and metabolic PAM studies.

## 1 Introduction

Photoacoustic microscopy (PAM) has become widely used in biomedical research due to its unique capability for microvascular imaging in a label-free and quantitative manner [1], [2], [3], [4], [5]. Leveraging the optical absorption of blood hemoglobin, PAM offers not only high image contrast but also exquisite sensitivity to blood oxygenation (sO_2_) and flow speed [6], [7], [8], [9], [10], [11], [12], [13]. Further, simultaneous imaging of these functional parameters, as achieved by multi-parametric PAM, has enabled quantification of the metabolic rate of oxygen, an important physiological parameter [10], [14]. As a result, PAM has been widely used to study hemodynamic and oxygen-metabolic responses under various physiological and pathological conditions, particularly in the brain [15], [16], [17], [18], [19], [20], [21], [22], [23], as well as other tissues or organs [24], [25], [26], [27], [28], [29], [30].

Among the functional parameters measured by PAM, sO_2_ is typically assessed through spectroscopic analysis. This method involves solving linear equations derived from measurements taken at two or more wavelengths of laser excitation, making it both straightforward and time-efficient [6], [7]. In contrast, the quantification of blood flow presents greater challenges in both accuracy and computational cost. Multiple methods have been developed for PAM-based flow quantification, but each has its limitations [31]. For example, the broadening of photoacoustic Doppler bandwidth has been exploited to quantify blood flow [9], [32], [33]; however, it requires averaging over thousands of measurements, and the measurable flow range is very limited. Another method relies on local heating and blood flow-induced thermal clearance [34], [35], [36]; however, it suffers from poor accuracy at low flow speeds and long acquisition time due to slow thermal decay. Moreover, some high-speed PAM systems use the kymograph method for blood flow measurements [13]; although providing accurate quantification of low flow speeds, it struggles to measure high speeds due to frame rate limitations. Additionally, although machine learning and deep learning-based methods have rapidly advanced in PAM research [37], [38], [39], [40], [41], [42], [43], [44], their contributions to blood flow quantification are mostly indirect—primarily through enhancing image quality and acquisition speed—rather than directly addressing flow quantification algorithms.

Currently, a correlation analysis-based approach, also known as correlation spectroscopy, is most widely used for blood flow imaging in PAM [6], [7], [8], [45], [46]. This technique collects successive photoacoustic signals from flowing red blood cells (RBCs), from which the correlation coefficients of the first and the subsequent signals are calculated. The correlation function follows a Gaussian decay, where the decay constant is linearly proportional to the blood flow speed. It has been demonstrated that this method can measure flow speeds in the range of 0.2–20 mm/s and has been applied by different PAM systems for *in vivo* studies [6], [7]. Although outperforming other PAM-based flow quantification methods, it has limitations in computational efficiency and accuracy. Correlation analysis and curve fitting are needed for flow quantification at each pixel, leading to prolonged data processing time that sometimes goes up to hours in order to generate a single image of blood flow [47]. The long computational time presents practical barriers to *in vivo* applications of functional PAM, especially in scenarios that involve the processing of large datasets, such as wide-field imaging [20], [22], [48], longitudinal monitoring [21], [23], [26], and large-cohort studies [14], [18], [19]. Moreover, the accuracy of this method is less reliable at high (i.e., >10 mm/s) and low (i.e., <1 mm/s) flow speeds, as shown in [6], [7]. This inaccuracy arises from non-ideal selections of the correlation time window (i.e., data acquisition window) and the presence of noise, which are often hard to avoid.

Here, we introduce a new frequency analysis-based approach for quantifying flow speed in PAM. While blood flow reduces the correlation between sequentially acquired photoacoustic A-line signals, it also modulates the amplitude of the signals. Such amplitude modulation can be leveraged to measure blood flow because its frequency is linearly proportional to the flow speed. Based on this, we have developed Hybrid Fourier-Derivative Analysis (HFDA) to accurately quantify the frequency of flow-induced amplitude modulation. By adaptive combination of the Fast Fourier Transform (FFT) and a derivative analysis, HFDA achieves accurate measurements across a broad range of blood flow speeds (0.2–20 mm/s), with most errors less than 7 %, and reduces the computational expense by 35 times compared to the traditional correlation analysis-based method. HFDA has been validated in both phantom and *in vivo* studies.

## 2 Methods

In photoacoustic imaging, the signal amplitude is determined by the fluence of the excitation light and the optical absorption of the object being imaged [1], [2], [5]. When imaging flowing blood, the amplitude of the signal is modulated periodically due to the movement of RBCs. In fast blood flow, RBCs rapidly move in and out of the illumination zone, resulting in high-frequency amplitude modulations. In contrast, slower blood flow produces lower-frequency modulations. By repeated imaging at the same location and analyzing the flow-induced amplitude modulations, the blood flow speed can be quantified.

**Fig. 1:**
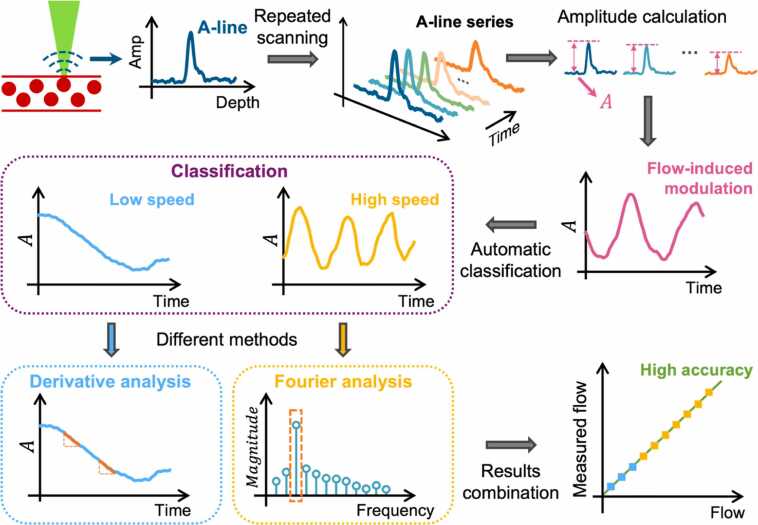
Schematic of Hybrid Fourier-Derivative Analysis for blood flow speed quantification in photoacoustic microscopy. A: amplitude.

Fig. 1 shows the working principle of the proposed HFDA method. We perform repeated scans at or near the same location, recording a series of photoacoustic A-line signals. Note that the data acquisition procedure differs slightly between the phantom and *in vivo* studies. For method validation and speed calibration in phantoms, A-lines are acquired exactly at the same location. In contrast, for *in vivo* imaging, dense raster scanning is utilized, with a step size much smaller than the size of RBCs, so A-lines are obtained from the vicinity of each location (further details are provided in Section 3). The signal amplitude is quantified as the maximum value of the Hilbert transformed A-line signal. By plotting the amplitudes of successive A-lines against time (or A-line index), a periodic modulation is observed, where the frequency corresponds to the blood flow speed. To extract the modulation frequency, we have developed the HFDA method. The idea of HFDA is to differentiate the amplitude modulations induced by high and low flow speeds and then to apply different analysis for each to ensure both high measurement accuracy and computational speed. The rationale behind the need for separate analyses, how they work, and how to adaptively combine them are detailed in 2.1, 2.2, 2.3. Section 2.4 details the phantom experiment for validating and calibrating HFDA prior to its application *in vivo*.

### 2.1 Fourier analysis of high-frequency modulation

As shown in the purple dashed box in Fig. 1, different blood flow speeds modulate the photoacoustic amplitude at different frequencies. Thus, the flow speed can be measured by analyzing the modulation frequency. A common approach for frequency analysis is the Fourier transform. Specifically, we perform the Fourier transform on the blood flow-modulated amplitude data to identify the main modulation frequency (highlighted in the yellow dashed box in Fig. 1), which is defined as the frequency component with the highest magnitude in the spectrum. Given the photoacoustic amplitudes $A\left(t\right)$ within the acquisition time window, the quantified main frequency $f_{m}$ can be expressed as:

(1) $$F\left(f\right)=F\left[A(t)\right],$$

(2) $$f_{m}=\underset{f}{\mathrm{argmax}}\left|F\left(f\right)\right|,$$

where t, f, and $F\left(f\right)$ represent the time, frequency, and Fourier transform of $A\left(t\right)$, respectively. Then, the blood flow speed can be derived from $f_{m}$ through a linear relationship, which can be calibrated using a phantom experiment.

To efficiently calculate the Fourier transform, we employ the FFT algorithm, which is well-known for its high computational efficiency. However, this method has limitations when dealing with slow blood flow. Low flow speeds generate low-frequency modulations, where a complete period of such slow modulation may not be captured due to the limited data acquisition time. As a result, the Fourier analysis cannot accurately identify the main frequency, leading to errors in flow speed measurements. Since data acquisition time is constrained by the need for fast imaging, a complementary data analysis method is required to accurately quantify slow blood flow.

### 2.2 Derivative analysis of low-frequency modulation

We propose a derivative analysis-based method to extract the low-frequency amplitude modulation caused by low blood flow speed. Given only a segment of the modulation period (the blue dashed box in Fig. 1), the rate of change in the amplitude within the segment provides an estimate of the modulation frequency. Specifically, slower modulation results in slower changes in the amplitude. The rate of change can be quantified by extracting the normalized absolute derivative of amplitude as:

(3) $$D(t_{i})=\frac{A(t_{i+1})-A(t_{i})}{t_{i+1}-t_{i}},$$

(4) $$D_{n}(t_{i})=\frac{\left|D(t_{i})\right|}{A(t_{i})},$$

in which i denotes the delay index (i.e., index of successive A-lines; i=1,2,…,M−1, M is the total number of acquired A-lines), $D(t_{i})$ denotes the estimated derivative of amplitude at $t_{i}$, and $D_{n}(t_{i})$ represents the normalized absolute derivative. Here, the absolute value ensures that positive and negative changes in the amplitude are treated equally. Normalizing the derivative by the amplitude prevents the rate of change from being affected by the original amplitude of the signal. Then, $D_{n}(t_{i})$ for each successive pair of A-lines within the segment is calculated, from which the mean is derived as:

(5) $$\overset{®}{D_{n}}=\frac{\sum_{i=1}^{M-1}D_{n}(t_{i})}{M-1}.$$

This averaging process effectively reduces variability in the derivatives within each cycle. Similarly to $f_{m}$, $\overset{®}{D_{n}}$ can be mapped to blood flow speed through a linear relationship, which can be calibrated using a phantom experiment. The calculation of $\overset{®}{D_{n}}$ is computationally efficient since it involves only basic operations. Note that this derivative-based approach is specifically effective for frequency analysis when only a portion of the modulation period is captured. For high-frequency modulation, where the data acquisition window typically contains multiple periods of modulation, applying the derivative analysis would lead to inaccurate measurements.

### 2.3 Hybrid Fourier-Derivative Analysis (HFDA)

Although we have developed two different analyses for the quantification of high and low flow speeds separately, proper selection and combination of them are crucial to achieving high accuracy across a broad range of blood flow. Therefore, HFDA is developed to combine Fourier and derivative analysis.

As illustrated in Fig. 1, HFDA first classifies the amplitude modulation as being induced by either high or low flow speed (the purple dashed box). Then, fast flow-induced modulation is processed using Fourier analysis (the yellow dashed box), while slow flow-induced modulation is analyzed using the derivative method (the blue dashed box). Finally, the flow speed is derived based on the measured $f_{m}$ or $\overset{®}{D_{n}}$, using the linear relationships validated and calibrated through the phantom experiments.

Proper classification of the amplitude modulation is essential for HFDA because applying Fourier analysis to low-frequency modulation or derivative analysis to high-frequency modulation can lead to significant measurement errors. The computational cost of the classification is another crucial factor, as it directly affects the overall efficiency of flow quantification. For *in vivo* studies, where the flow speed can be either high or low, features of the amplitude modulation and results from the two different analyses are leveraged for classification. Based on observations from the phantom study and considerations for computational efficiency, we use the result of the derivative analysis as the criterion for classification in HFDA. Specifically, we first apply derivative analysis to each amplitude modulation, checking if the measured flow speed exceeds a predefined threshold. If so, the data is classified into the fast-flow category and reprocessed using Fourier analysis, otherwise, the data is assigned into the slow flow category, and the result from prior derivative analysis is considered accurate. This classification method has shown robust performance while minimizing additional computations, as detailed in Section 3.

### 2.4 Phantom experiment and calibration

**Fig. 2:**
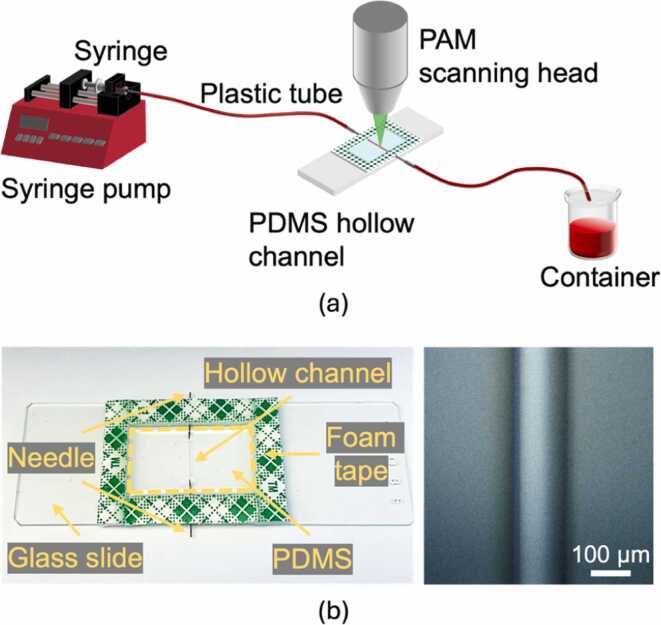
Phantom study for HFDA validation and calibration. (a) Schematic of the experimental setup. (b) Camera (left) and microscope (right) photos of the PDMS-based hollow channel.

A phantom study is required for the validation and calibration of HFDA. Fig. 2a shows the experimental setup used for this study. The phantom consists of a microfluidic channel made of polydimethylsiloxane (PDMS), with defibrinated bovine blood (910, Quad Five) flowing through it. A syringe pump (NE-300, New Era Pump Systems Inc.) is utilized to generate a constant blood flow at preset speeds. The syringe (309659, BD) on the pump is connected to the microfluidic channel via a plastic tube (AAD04091, United States Plastic Corporation). The flowing blood is imaged by the PAM scanning head (laser spot: 3.4 µm) fixed at the center of the channel. The PDMS hollow channel is custom-designed and fabricated, with a 127-µm diameter (Fig. 2b). The PDMS (Sylgard 184, Electron Microscopy Sciences) is chosen due to its high optical transparency and low acoustic loss [49], [50]. Two 30-gauge syringe needles (305106, BD) are modified to connect the channel to external plastic tubes. The used blood is collected by a container at the distal end. Below is the step-by-step fabrication process of the PDMS channel:

**Step 1.** Insert a 127-µm copper wire (8873K36, McMaster-Carr) into two modified 30-gauge syringe needles. The modification of needles involves removing the plastic wrap in the syringe needles and uncovering both sides of the needle.

**Step 2.** Mount the copper wire and modified syringe needles on a glass slide (1321, Globe Scientific) using foam tapes (4032, 3M). Ensure that the copper wire is straight. The 1-mm-thick foam tapes form a well to house PDMS.

**Step 3.** Prepare PDMS by mixing the curing agent and polymer base (1:5) and diluting it with hexane (1:1).

**Step 4.** Apply PDMS into the well to cover both the copper wire and the needles. Cure it at 100 °C and <0.1 inHg for 60 min in a vacuum oven (AT09p7.110, Across International). Then, cool down at room temperature for 120 min.

**Step 5.** Carefully remove the copper wire, resulting in the formation of a hollow channel that connects the two needles.

The HFDA validation and calibration is performed as follows. First, data acquired in the blood-flowing phantom are processed using both Fourier and derivative analysis. This allows for the examination of the linear relationship between the preset flow speeds and the experimentally measured parameters (i.e., $f_{m}$ in Fourier analysis and $\overset{®}{D_{n}}$ in derivative analysis). The parameters for mapping $f_{m}$ and $\overset{®}{D_{n}}$ to blood flow speed are then calibrated through linear regression. After calibration, the same phantom data are processed using HFDA, and the flow speeds derived by HFDA are compared to the preset values to validate its accuracy. Additionally, the computational efficiency of all the methods—Fourier analysis, derivative analysis, HFDA, and the traditional correlation analysis—is quantified and compared.

## 3 Results

First, we validated and calibrated the two frequency analyses for blood flow quantification in phantoms. In this study, blood flowing through the PDMS channel was continuously imaged by the PAM system. The time interval between adjacent A-lines was 0.15 ms. The preset flow speed ranged from 0.2 to 20 mm/s, with different intervals for different speed ranges: a 0.2-mm/s interval for the 0.2–1 mm/s range, a 1-mm/s interval for the 1–2 mm/s range, and a 2-mm/s interval for the 2–20 mm/s range. For each preset flow speed, 10 individual groups of data were acquired, with each group consisting of 14,400 A-lines. A time window of 100 successive A-lines was used for data processing. Variations in laser fluence, which could affect the amplitude of A-lines, were compensated for by using a photodiode (PDA36A2, Thorlabs) to monitor the laser fluctuation.

**Fig. 3:**
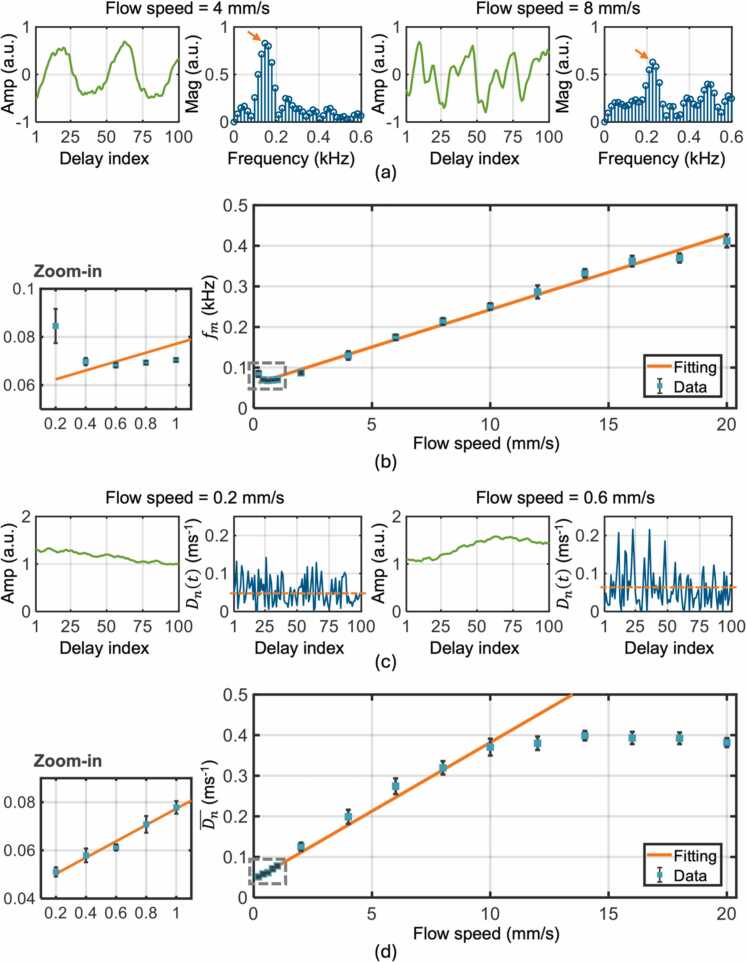
Phantom validation of Fourier analysis-based and derivative analysis-based flow speed quantification. (a) Examples of high-frequency photoacoustic amplitude modulation and Fourier analysis. Left: 4 mm/s. Right: 8 mm/s. The orange arrows indicate the measured main frequencies $f_{m}$. The delay index also represents the A-line number, with a time interval of 0.15 ms between adjacent A-lines. Amp: amplitude. Mag: magnitude. (b) Phantom results and calibration of the Fourier analysis-based method. Fitting range: 1–20 mm/s, R^2^: 0.9911. (c) Examples of low-frequency photoacoustic amplitude modulation and derivative analysis. Left: 0.2 mm/s. Right: 0.6 mm/s. The orange dashed lines represent the measured mean normalized absolute derivatives $\overset{®}{D_{n}}$. (d) Phantom results and calibration of the derivative analysis-based method. Fitting range: 0.2–1 mm/s, R^2^: 0.9818.

**Figure A.1:**
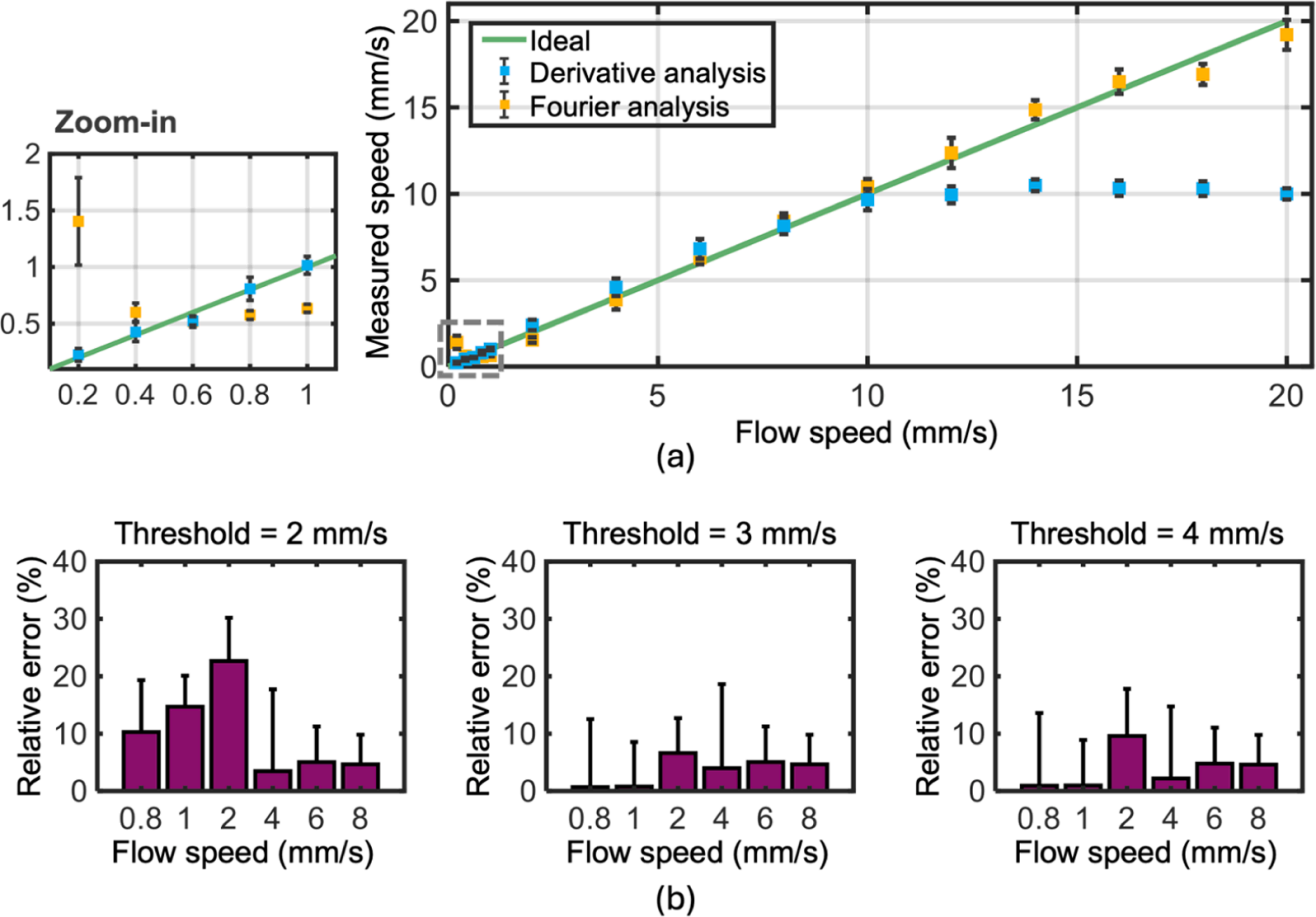
Flow speed classification in HFDA. (a) Phantom results of Fourier and derivative analysis after converting measured $f_{m}$ and $\overset{®}{D_{n}}$ to speed. The green line represents the ideal result, not a fitting curve. (b) Relative measurement errors in the medium range of flow speeds when using different classification thresholds. Left: threshold set to $\overset{®}{D_{n}}$ corresponding to a measured speed of 2 mm/s. Middle: threshold set to $\overset{®}{D_{n}}$ corresponding to a measured speed of 3 mm/s. Right: threshold set to $\overset{®}{D_{n}}$ corresponding to a measured speed of 4 mm/s.

The phantom data were processed by Fourier and derivative analysis separately, and the results are shown in Fig. 3a-b and Fig. 3c-d, respectively. Figs. 3a and 3c present examples of high- and low-frequency photoacoustic amplitude modulations induced by high and low flow speeds, respectively, along with corresponding Fourier and derivative analysis. In the high flow speed data (Fig. 3a), multiple cycles of amplitude increase and decrease are observed within the time window. In contrast, the low flow speed data (Fig. 3c) shows only a segment of such a cycle. Moreover, the measured $f_{m}$ (indicated by arrows in Fig. 3a) and $\overset{®}{D_{n}}$ (indicated by dashed lines in Fig. 3c) vary with the preset flow speeds. The mean values of $f_{m}$ and $\overset{®}{D_{n}}$ are plotted against the preset flow speeds in Figs. 3b and 3d, respectively. For the Fourier analysis, the linear relationship between $f_{m}$ and medium-to-high flow speeds (1–20 mm/s) is confirmed with a high R^2^ value of 0.9911. Similarly, for the derivative analysis, the linear relationship between $\overset{®}{D_{n}}$ and low-to-medium speeds (0.2–1 mm/s) shows an R^2^ value of 0.9818. Besides validation, the linear regression also serves as a phantom calibration, which determines the parameters required for converting $f_{m}$ and $\overset{®}{D_{n}}$ into measured flow speeds. The measured flow speeds against corresponding preset values are plotted in Appendix Fig. A.1a.

**Fig. 4:**
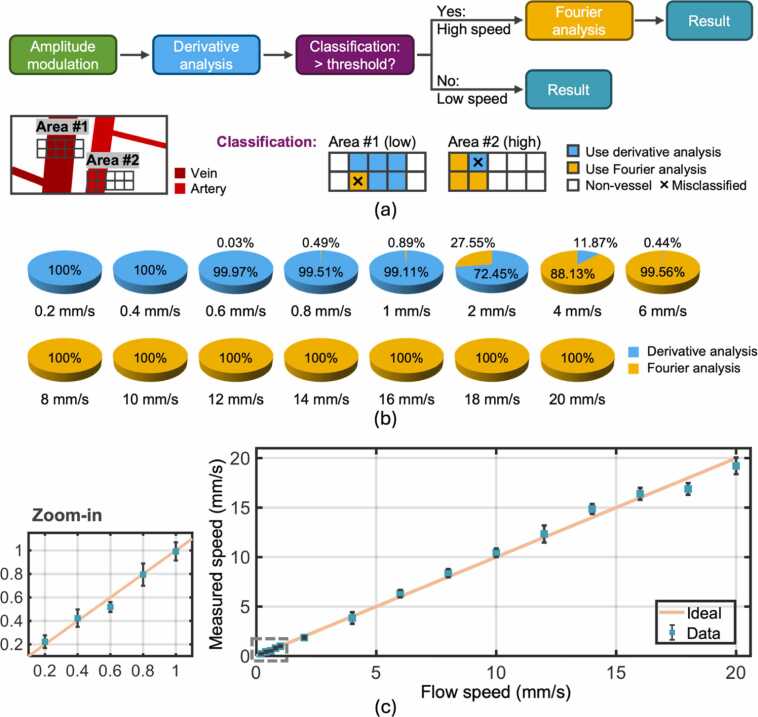
Phantom validation of HFDA-based flow speed quantification. (a) Data processing workflow of HFDA, with a detailed illustration of the classification step shown in the bottom schematic. (b) Classification results obtained using HFDA. (c) Phantom results obtained using HFDA. The peach line, included as a reference, represents the ideal result where the measured speed equals the preset speed. The relative error at each preset speed is provided in Appendix Table B.1.

After validating and calibrating the two analyses for separate quantification of high and low flow speeds, we validated HFDA using the same phantom data. The need for HFDA is apparent from Fig. 3b and Fig. 3d, which show poor linear relationships between $f_{m}$ and low flow speeds (<1 mm/s) and between $\overset{®}{D_{n}}$ and high flow speeds (>4 mm/s). Fig. 4a shows the workflow of HFDA, with the detailed process of flow speed classification. As introduced in Section 2.3, HFDA begins with the derivative analysis of amplitude modulation, checking if the measured $\overset{®}{D_{n}}$ is larger than the threshold to classify the modulation as being induced by either a high or low flow speed. As shown in Fig. 3d, the linear relationship between $\overset{®}{D_{n}}$ and flow speed is invalid when the speed becomes too high. This saturation effect makes it suitable to distinguish high speeds from low speeds based on a threshold of $\overset{®}{D_{n}}$. If the amplitude modulation is assigned to the high-speed category after classification, Fourier analysis is used to re-quantify the flow speed.

The classification threshold is defined based on the phantom results obtained from Fourier and derivative analysis. As shown in Appendix Fig. A.1a, both methods yield accurate quantification of flow speeds within the range of 2–4 mm/s. To minimize the impact of misclassification, which is typically unavoidable near the threshold, we should select a $\overset{®}{D_{n}}$ value that corresponds to a flow speed in this range. As illustrated in the bottom row of Fig. 4a, pixels in the low-speed area might be misclassified as high speed, and vice versa. However, such misclassification does not significantly affect the measurement accuracy in the flow speed range of 2–4 mm/s, where both analyses exhibit high accuracy. We tested different $\overset{®}{D_{n}}$ values corresponding to the flow speeds of 2, 3, and 4 mm/s, respectively, as the classification threshold and compared the measurement errors. As shown in Appendix Fig. A.1b, the $\overset{®}{D_{n}}$ value corresponding to 3 mm/s resulted in the smallest overall measurement error and is thus selected as the classification threshold.

The classification method was tested using the phantom data, and the accuracy was assessed by dividing the number of pixels correctly assigned to either Fourier or derivative analysis by the total pixel number. As shown in Fig. 4b, the misclassification rate is less than 1 % in both low (<1 mm/s) and high (>4 mm/s) speed ranges.

After confirming the reliability of the classification process, the entire HDFA process was performed on the phantom data. The flow speeds measured using HFDA were compared against the preset values, and the errors of corresponding measurements were quantified. The high accuracy of HFDA is confirmed by that the measured flow speeds are closely aligned with the true values (Fig. 4c), with most of the errors less than 7 % (Appendix Table B.1).

**Fig. 5:**
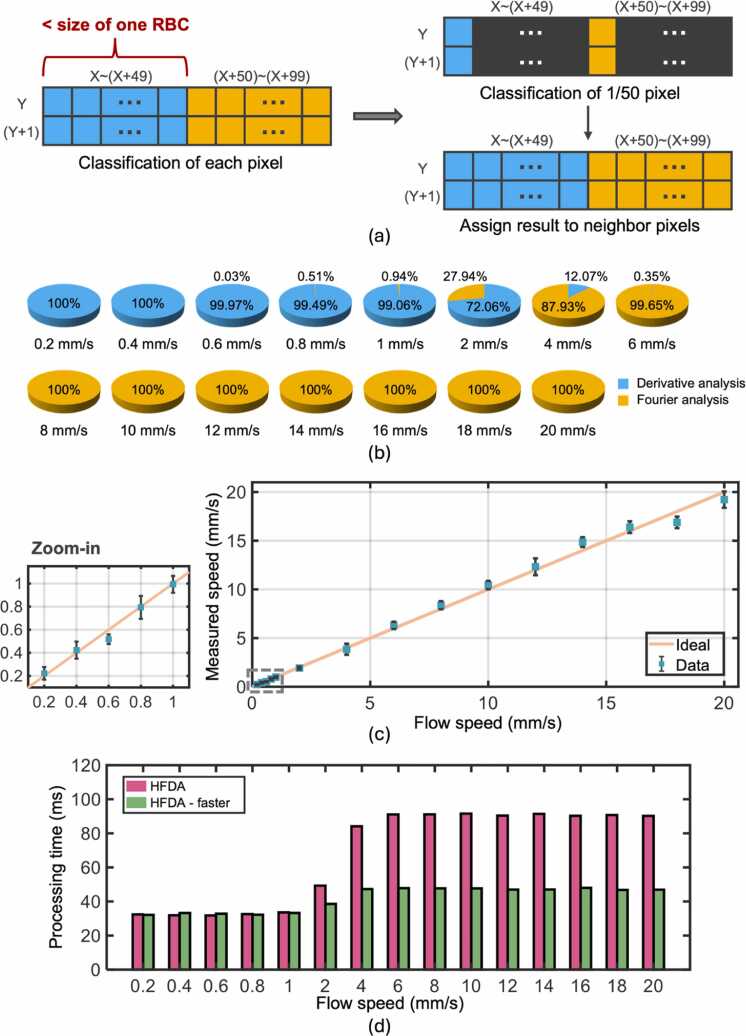
Phantom validation of the faster implementation of HFDA. (a) Comparison of the classification strategies used in the original HFDA (left) and the faster implementation of HFDA (right). (b) Classification results from the faster implementation of HFDA. (c) Phantom results from the faster implementation of HFDA. The peach line represents the ideal result, not a fitting curve. The relative error for each preset speed is provided in Appendix Table B.1. (d) Average data processing time for each preset speed using the original HFDA (pink bar) and the faster implementation of HFDA (green bar).

In addition to the measurement accuracy, the computational efficiency of HFDA was evaluated by quantifying the average time of data processing performed in MATLAB R2023b on a computer equipped with an Intel(R) Xeon(R) W-2275 CPU @ 3.30 GHz. No parallel or GPU computing was used. As shown in Appendix Table B.2, both Fourier and derivative analysis yield high computational speeds. Although slower than them, HFDA achieves a more than 20-fold improvement over the traditional correlation analysis. The slower performance of HFDA is due to the redundant analysis of high flow speed-induced amplitude modulations (i.e., classification with derivative analysis and re-quantification with Fourier analysis). To address this issue, we implemented a more efficient version of HFDA, termed HFDA-faster, in which only a subset of imaging points is analyzed twice. Given that the total scanning distance of 50 pixels is less than the size of a single RBC, the flow speed is unlikely to vary significantly. Therefore, only every 50th pixel is classified using derivative analysis, and the classification result is applied to the 49 neighboring pixels, as shown in Fig. 5a. This approach allows for direct selection of the correct category for 98 % of the data, without redundant derivative analysis. HFDA-faster was tested on the same phantom data, with the results shown in Fig. 5b-c and Appendix Table B.1-B.2. The minimal differences in both measured flow speeds (Fig. 4b-c *vs.* Fig. 5b-c) and measurement errors (Appendix Table B.1) between HFDA and HFDA-faster validate this faster implementation. Notably, HFDA-faster reduces processing time by 40 % (Fig. 5d), achieving a 35-fold improvement in computational speed over the correlation analysis-based method (Appendix Table B.2).

Finally, HFDA was applied for blood flow imaging *in vivo*. The same PAM system was used for data acquisition, and raster scanning was performed with a fine motor step size of 0.10 µm [6], [8], [26]. It should be noted that since scanning is involved, the measured speed is the resultant speed of blood flow speed and motor speed, which is set to 0.67 mm/s in our system. To extract the true blood flow speed, we employed a bi-directional scanning strategy [6], [9]. Additionally, HFDA was modified to address a vessel boundary issue encountered when measuring blood flow in microvessels with small diameters. The *in vivo* demonstration was conducted in established mouse models of hypoxia and hypercapnia. During imaging, CD-1 mice (female, 12 weeks old, Charles River Laboratories) were anesthetized with 1.5 % isoflurane, and the body temperature was maintained at 37 °C using a temperature-controlled heating pad. All animal experiment procedures involved in this study were conducted in compliance with the laboratory animal protocol approved by the Institutional Animal Care and Use Committee at Washington University in St. Louis.

**Fig. 6:**
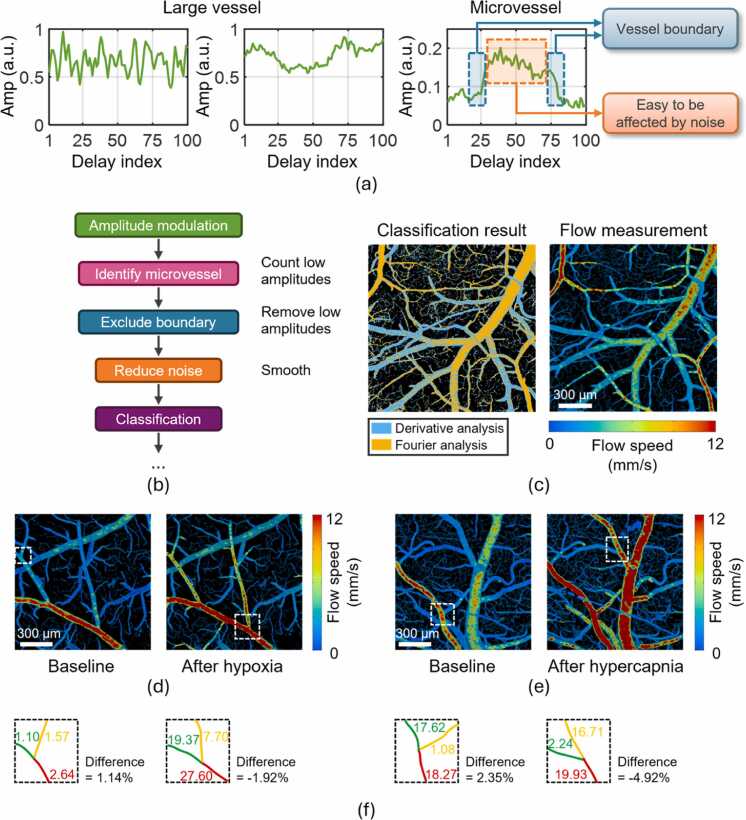
Application of HFDA for PAM-based blood flow imaging *in vivo*. (a) Examples of photoacoustic amplitude modulation in large vessels and microvessels. Left: high-frequency modulation in large vessels. Middle: low-frequency modulation in large vessels. Right: low-frequency modulation in microvessels. (b) Modified workflow of HFDA for pre-processing of *in vivo* data. Starting from classification, subsequent processing follows the same steps as those used in phantom experiments. (c) Quantification of the flow speeds in mouse cerebral vessels using the faster implementation of HFDA. Left: classification result. Right: flow speed map. FOV: 1.5 × 1.5 mm^2^. (d) Flow speed images of mouse cerebral vessels at baseline and after hypoxia (12 % O_2_). FOV: 1.2 × 1.2 mm^2^. (e) Flow speed images of mouse cerebral vessels at baseline and after hypercapnia (10 % CO_2_). FOV: 1.2 × 1.2 mm^2^. (f) Blood flow conservation at vascular bifurcation. The bifurcation sites are selected from the regions marked by the white dashed boxes in (d) and (e). Unit: nL/s. The difference (%) represents the relative error between the upstream flow (red) and the combined downstream flow (green and yellow).

Fig. 6a presents *in vivo* examples of blood flow-induced photoacoustic amplitude modulation and highlights the need for modifying the data processing in HFDA for *in vivo* applications. While both high-frequency and low-frequency modulations are clearly observed in large vessels, low-frequency modulations in microvessels are obscured by abrupt amplitude changes caused by vessel boundaries. Moreover, microvessels produce weaker photoacoustic signals, making the amplitude modulation more susceptible to noise. These factors contribute to inaccuracies in the measurement of microvascular flow speed. To address these issues, we incorporated the following pre-processing steps into the HFDA workflow (Fig. 6b): (1) examine if the data are from microvessels by counting the number of low amplitudes in the data acquisition window; (2) exclude vessel boundary-induced amplitude changes by removing low-amplitude data; (3) reduce noise by smoothing the amplitude modulation data. After pre-processing, classification is applied, followed by the subsequent steps in HFDA.

**Figure A.2:**
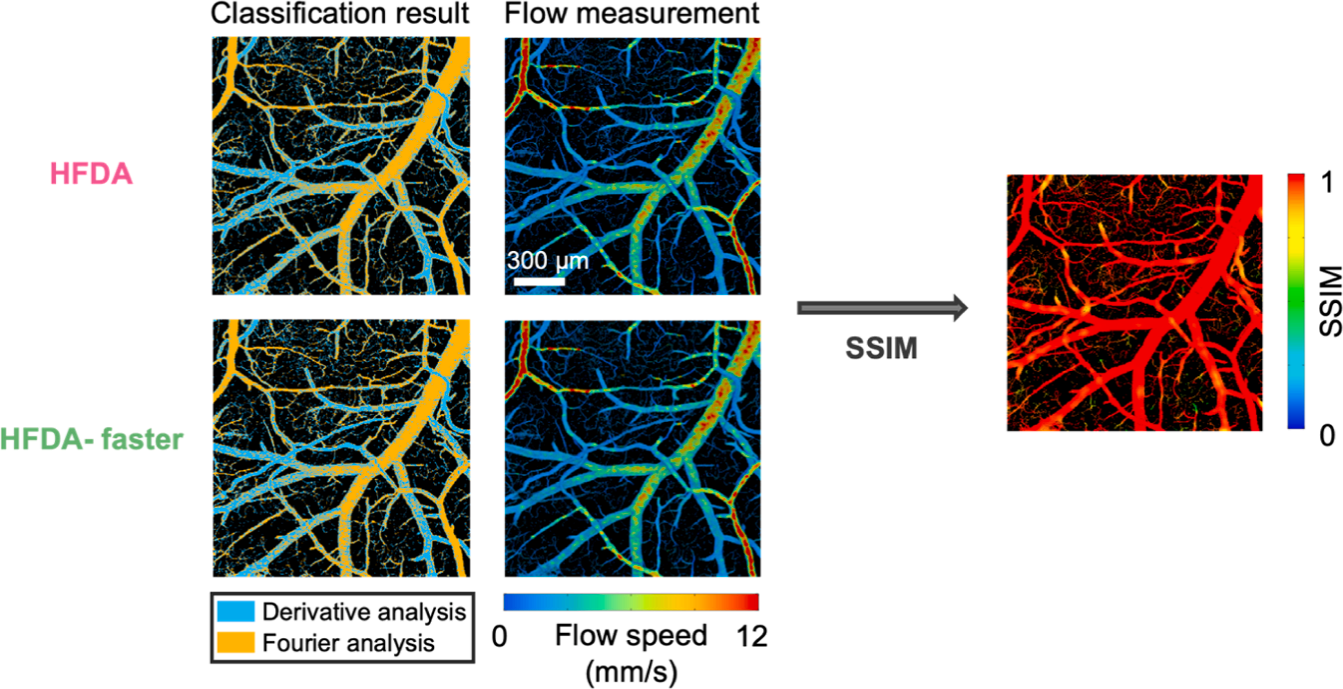
Validation of the faster implementation of HFDA *in vivo*. Top left: classification result and flow map obtained using the original HFDA. Bottom left: classification result and flow map obtained using the faster implementation of HFDA (the same as Fig. 6c). Right: pseudocolor-coded SSIM map, with a mean SSIM value of 0.95. FOV: 1.5 × 1.5 mm^2^.

Using the modified HFDA and its faster implementation, a pseudocolor-encoded map of the blood flow speed in the mouse brain was obtained (Fig. 6c). The left panel of Fig. 6c shows the classification result, where high-speed and low-speed areas are accurately assigned to Fourier analysis and derivative analysis, respectively. The right panel presents the final flow map, generated by spatially averaging HFDA-measured flow speeds within each 5 × 5 μm² region and applying a customized colormap for visualization. To validate the faster implementation of HFDA *in vivo*, the same data were processed using both the standard and faster versions of HFDA. As shown in Appendix Fig. A.2, a pixel-by-pixel comparison of the flow measurement results obtained from the two versions of HFDA yields a Structural Similarity Index Measure (SSIM) value of 0.95, indicating near-identical performance *in vivo*.

Also, the computational efficiency of HFDA was assessed *in vivo*, to determine the impact of the extra pre-processing steps in the modified HFDA. As shown in Appendix Table B.3, the pre-processing caused negligible slowdown (<0.3 %), and the faster version of HFDA is 33 times faster than the correlation analysis when processing *in vivo* data.

After validating its accuracy and efficiency, we demonstrated the use of HFDA by quantifying the responses of cerebral blood flow to hypoxia (by mixing nitrogen with medical air to lower the O_2_ concentration to 12 %) and hypercapnia (by mixing 10 % CO_2_ with medical air), both of which are established models known to increase blood flow speed in the mouse brain [8], [17], [48]. The blood flow speed measured by HFDA under baseline conditions and following hypoxia and hypercapnia are shown in Fig. 6d and Fig. 6e, respectively. Significant flow increases are observed in cerebral vessels in response to both challenges, confirming that HFDA can be applied to functional PAM studies *in vivo*. To further validate the accuracy and reliability of *in vivo* blood flow measurements using HFDA, we selected four vascular bifurcation sites from the images in Fig. 6d-e (indicated by the white dashed boxes) and assessed whether the total blood flow volume remains conserved before and after each bifurcation [9], [24], [51]. As shown in Fig. 6f, blood flow volumes in the upstream (red) and downstream (green and yellow) branches were quantified based on HFDA-measured flow speeds and vessel diameters. The relative differences in total flow before and after bifurcation at all selected sites were less than 5 %, further supporting the reliability of HFDA for *in vivo* blood flow measurement.

## 4 Conclusion and discussion

In summary, we have developed the Hybrid Fourier-Derivative Analysis for the quantification of blood flow speed in PAM. It analyzes the frequency of photoacoustic amplitude modulation induced by blood flow, enabling accurate (with most errors less than 7 %) and fast (35-fold faster than the traditional correlation analysis) flow measurement within a wide physiological range (0.2–20 mm/s). The effectiveness of HFDA has been validated through both phantom and *in vivo* studies.

With high computational speed and low complexity, HFDA holds the potential to enable real-time blood flow quantification during image acquisition. To this end, the computational speed of HFDA can be further enhanced with acceleration strategies, such as parallel computing and GPU processing. The substantially reduced computational time alleviates practical challenges in the applications of functional PAM that demand high-throughput data processing, such as wide-field or longitudinal imaging and large-cohort studies [14], [18], [19], [20], [21], [22], [23], [26], [48]. The ability to visualize results in real time or shortly after data acquisition provides researchers with immediate feedback, facilitating rapid detection of unexpected findings and timely adjustments to experimental protocols. In the long term, the improved computational efficiency could also facilitate the clinical translation of PAM, where delays in data processing can impede diagnosis, decision-making, and interventions. Additionally, HFDA significantly lowers data storage requirements for blood flow measurements. Unlike current methods such as the correlation analysis, which save the entire A-line for analysis, HFDA only requires storing the amplitude of the A-line. This is particularly important when handling large three-dimensional datasets, where efficient data storage and processing are crucial.

HFDA holds significant promise for advancing PAM studies on vascular hemodynamics, owing not only to its high accuracy and efficiency but also its ease of implementation. Unlike existing fast flow measurement techniques—such as the GPU-accelerated correlation-based method [47]—which relies heavily on GPU and CUDA programming, HFDA achieves high computational efficiency without the need for high-performance computing hardware or advanced programming skills, thereby enhancing its accessibility. Moreover, HFDA is compatible with simple raster scan-based data acquisition and requires only standard signal processing techniques (e.g., FFT) for data analysis. By following the phantom calibration detailed herein, HFDA can be readily adapted to different PAM systems.

Although HFDA is not specific to a particular PAM design, it does have requirements on the system’s signal-to-noise ratio (SNR) and data acquisition. The SNR must be sufficiently high to distinguish the flow-induced amplitude modulation from noise. When the SNR falls below 2, significant deviations can occur, leading to unreliable measurements, especially for low flow speeds that rely on the derivative analysis. Regarding data acquisition, the A-line rate (i.e., sampling rate) should be sufficient to capture the rapid amplitude modulation induced by a high flow speed. Additionally, the data acquisition window (i.e., the number of repeatedly acquired A-lines) must be long enough to record detectable amplitude modulations by the low flow speed.

Currently, HFDA focuses on improving the speed of blood flow data processing rather than acquisition in PAM. However, HFDA does show potential in this regard. The speed of blood flow data acquisition is primarily limited by the slow amplitude modulation induced by the low flow speed. Using the derivative analysis in HFDA, this limitation is partially mitigated because an incomplete cycle of amplitude modulation can still be used to measure low flow speeds. The next step is to investigate the potential of applying HFDA to reduce the data acquisition time for PAM-based blood flow imaging.

## Funding

This work was supported in part by the 10.13039/100000002National Institutes of Health under Grant NS099261, NS120481, AG079503, and NS125677 and the 10.13039/100000001National Science Foundation under Grant 2023988.

## CRediT authorship contribution statement

**Zhuoying Wang:** Writing – review & editing, Writing – original draft, Visualization, Validation, Software, Project administration, Methodology, Investigation, Formal analysis, Data curation, Conceptualization. **Ziang Feng:** Writing – review & editing, Writing – original draft, Methodology, Investigation, Conceptualization. **Song Hu:** Writing – review & editing, Writing – original draft, Supervision, Resources, Project administration, Methodology, Investigation, Funding acquisition, Conceptualization.

## Declaration of Competing Interest

The authors declare the following financial interests/personal relationships which may be considered as potential competing interests: Given his role as Editor of Photoacoustics, Dr. Song Hu had no involvement in the peer review of this article and had no access to information regarding its peer review. Full responsibility for the editorial process for this article was delegated to another journal editor. Other authors declare that they have no known competing financial interests or personal relationships that could have appeared to influence the work reported in this paper.

## Figures and Tables

**Table B.1:** Relative measurement error of HFDA on phantom data

Flow speed (mm/s)	HFDA (%)	HFDA-faster (%)
0.2	11.1489	11.1489
0.4	5.8376	5.8376
0.6	13.7015	13.7002
0.8	0.7049	0.8106
1	0.8014	0.6725
2	6.6164	3.9002
4	3.9888	3.6974
6	5.0611	5.0335
8	4.6580	4.6580
10	4.4072	4.4072
12	2.7489	2.7489
14	6.1548	6.1548
16	2.4724	2.4724
18	6.1911	6.1911
20	3.8901	3.8901

**Table B.2:** Computational efficiency of different flow quantification methods on phantom data

Approach	Average processing time^a^ (ms)
Fourier analysis	44.49
Derivative analysis	32.13
HFDA	68.16
HFDA-faster	41.89
Correlation analysis^b^	1472.91

a The time is for processing 3600 quantifications.

b The correlation analysis-based method is performed on the same data with the same settings (e.g., time window).

**Table B.3:** Computational efficiency of different flow quantification methods on *in vivo* data

Approach	Average processing time^a^ (ms)
HFDA-faster	45.54
HFDA-faster + Microvessel	45.62
Correlation analysis^b^	1522.22

a The time is for processing 3600 quantifications.

b The correlation analysis-based method is performed on the same data with the same settings (e.g., time window).

## Data Availability

The datasets (i.e., phantom, *in vivo*) are not currently deposited into a public repository due to the large size. All data and code involved in this study are available from the corresponding author upon request.
